# Exploring mental health in veterinary students: common stressors and effective coping strategies: a narrative review

**DOI:** 10.3389/fvets.2025.1470022

**Published:** 2025-02-11

**Authors:** Rahib K. Islam, Emily K. Cobb, Hannah K. McCowan, Kylie Watson, Kaustuv Bhattacharya, Anjali Chandra, Warda Mohiuddin, Karen Gruszynski, Amanda H. Wilkerson, John J. Dascanio, Robert E. Davis, Vinayak K. Nahar

**Affiliations:** ^1^LSU Health Sciences Center New Orleans School of Medicine, New Orleans, LA, United States; ^2^Department of Pathology, University of Alabama at Birmingham, Birmingham, AL, United States; ^3^Department of Dermatology, School of Medicine, University of Mississippi Medical Center, Jackson, MS, United States; ^4^Center for Animal and Human Health in Appalachia, College of Veterinary Medicine, Lincoln Memorial University, Harrogate, TN, United States; ^5^Department of Pharmacy Administration, School of Pharmacy, University of Mississippi, University, MS, United States; ^6^Center for Pharmaceutical Marketing and Management, University of Mississippi School of Pharmacy, University, MS, United States; ^7^Brigham and Women Hospital, Boston, MA, United States; ^8^William Carey University College of Osteopathic Medicine, Hattiesburg, MS, United States; ^9^College of Graduate Studies, Midwestern University, Downers Grove, IL, United States; ^10^Department of Health Science, College of Human Environmental Sciences, The University of Alabama, Tuscaloosa, AL, United States; ^11^Texas Tech University School of Veterinary Medicine, Amarillo, TX, United States; ^12^Substance Use and Mental Health Laboratory, Department of Health, Human Performance and Recreation, University of Arkansas, Fayetteville, AR, United States; ^13^Department of Preventive Medicine, School of Medicine/John D. Bower School of Population Health, University of Mississippi Medical Center, Jackson, MS, United States

**Keywords:** veterinary students, mental health, stress, anxiety, depression, coping strategies, gender differences, social support

## Abstract

**Introduction:**

Veterinary students face significant challenges impacting their mental health and wellbeing. The rigorous academic curriculum, high expectations, and demanding clinical training create an environment of intense pressure and constant stress. This review explores the demographic and psychosocial variables influencing mental health outcomes, highlighting common stressors and coping strategies.

**Methods:**

A systematic literature search was conducted using PubMed, CAB Abstracts, and Google Scholar. Studies published up to 2021 involving veterinary students were included. Twenty-one peer-reviewed studies met the inclusion criteria. Demographic data, stressors, mental health measures, and coping strategies were extracted and analyzed. Studies focused on various mental health aspects, including stress, anxiety, depression, and coping mechanisms. The final selection was based on relevance, quality, and comprehensiveness.

**Results:**

The reviewed studies indicated that rigorous academic demands, high expectations, and clinical training contribute to elevated stress levels among veterinary students. Women reported higher levels of stress, anxiety, and depression compared to men, necessitating gender-specific interventions. Social support and relationship quality were crucial for mental wellbeing, with students in supportive environments experiencing lower stress. Effective coping strategies included exercise and social activities; whereas, harmful behaviors like substance misuse exacerbated mental health issues. Despite using mental health services, barriers such as stigma and lack of awareness hindered access for some students.

**Conclusion:**

The findings underscore the need for targeted support systems to enhance the mental health and resilience of veterinary students. Interventions to promote healthy coping mechanisms, reduce stigma, and improve access to mental health resources are essential. Veterinary schools should prioritize creating a supportive environment to ensure students can manage the demands of their profession while maintaining their mental health and wellbeing.

## Introduction

Veterinary students face unique and substantial challenges that significantly impact their mental health and wellbeing. Mental health refers to the presence or absence of mental illness and reflects the overall psychological state of the individual. On the other hand, wellbeing is a broader construct that includes positive aspects of mental and physical health, as well as a holistic state of flourishing characterized by life satisfaction and a sense of purpose.

For veterinary students, the rigorous academic curriculum, high expectations, and demanding clinical training create an environment of intense pressure and constant stress impacting mental health and wellbeing (1). Furthermore, there are many more conditions and factors to be aware of. Significant debt load, dealing with family issues while undergoing a rigorous curriculum, and even having cultural obligations are only a few factors that may be causes of stress. Unlike their peers in other academic disciplines, veterinary students must navigate the complexities of both medical and surgical knowledge, along with the emotional demands of caring for animals and interacting with pet owners (2). Consequently, veterinary students often experience elevated levels of stress, anxiety, and depression (3).

The prevalence of mental health issues among veterinary students is alarmingly high, as studies have consistently shown that these students report higher levels of perceived stress, anxiety, and depression compared to the general population (4). Numerous studies have identified several common stressors that impact veterinary students’ mental health. Academic demands, time pressure, and the challenging nature of the veterinary curriculum are frequently cited as significant sources of stress (5). Additionally, social factors such as homesickness, relationship status, social support, and living arrangements play a vital role in students’ psychological wellbeing (6). Gender differences have also been observed, with female students often reporting higher levels of stress and anxiety compared to their male counterparts (7).

Given the multifaceted nature of these challenges, it is imperative to understand the various factors contributing to mental health issues among veterinary students. This review explores the demographic and psychosocial variables that influence mental health outcomes, highlights common stressors such as academic workload and homesickness, and examines coping strategies employed by students. Additionally, it discusses the barriers to accessing mental health services and the effectiveness of different support mechanisms. By providing a comprehensive overview of the current state of mental health among veterinary students, this review seeks to inform educators, mental health professionals, and policymakers. It emphasizes the need for targeted interventions and support systems to mitigate stress, promote resilience, and improve the overall mental health and wellbeing of veterinary students.

## Methods

An array of study criteria were incorporated to ensure a diverse variety of studies were included and all studies used fit within the overall research objective. Inclusion criteria were studies where participants were U.S. veterinary students at an accredited veterinary college and measured an aspect of mental health as a research outcome. Literature searches included both English and non-English studies, and no restriction was placed on publication date. All studies were peer-reviewed and contained full data sets for evaluation. Studies were screened to remove duplicates, ongoing studies, conference abstracts, clinical studies, and studies that did not pertain to the research study group (i.e., veterinary students), or research study content (i.e., mental health).

Literature searches were performed at three separate times—June of 2019 and then again during June of 2020 and June of 2021 to capture studies from the year 2019–2021. Searches were conducted on the PubMed and CAB Abstracts databases as well as the Lincoln Memorial University Library and Google Scholar search engines. Varying combinations of the following keywords were used to screen for relevant studies: “veterinary student,” “veterinary school,” “mental health,” “depression,” “emotion,” “stress,” “anxiety,” and “depression.” All studies were reviewed by three independent reviewers.

**Figure 1:**
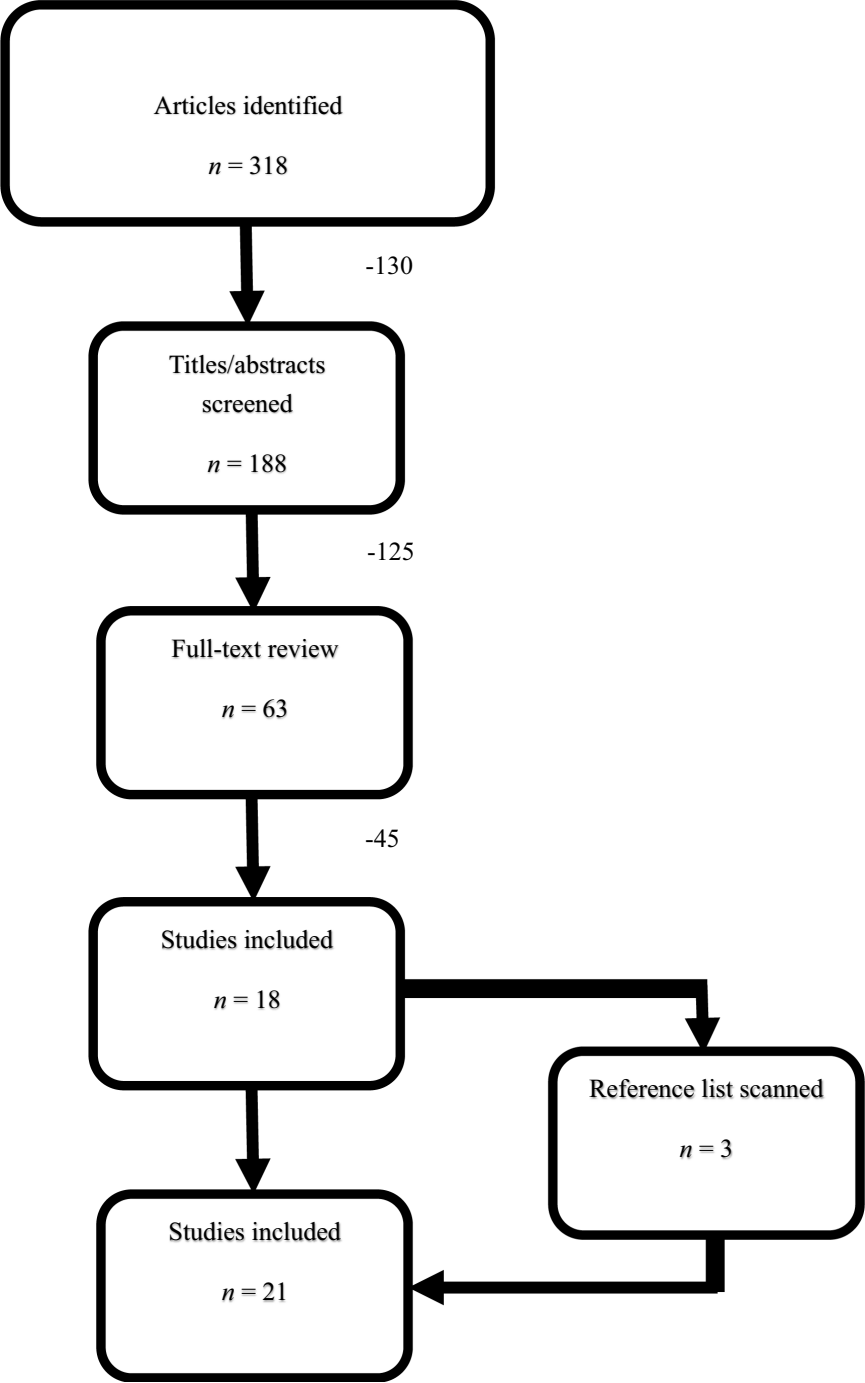
Literature search process.

The original list of publications was shortened by removing duplicate studies, irrelevant or incomplete studies, and studies that were not peer-reviewed. The remaining studies were screened in full-text to ensure all inclusion criteria was met. All discrepancies regarding meeting inclusion criteria were discussed among the three independent research reviewers until a decision could be reached about study inclusion. Once an exhaustive list of publications was formed and reviewed, researchers extracted data from each individual study for comparison. The extracted data was reviewed by all three independent researchers to ensure the data was correct and relevant to the overall research purpose. Once researchers reviewed and eliminated studies that did not meet the inclusion criteria, 21 studies remained for full analysis and data extraction. Figure 1 illustrates the flow diagram outlining the literature search procedure.

## Results

### Sample size and demographics

A total of 21 studies focusing on the mental health of veterinary students were reviewed, encompassing a wide range of sample sizes and demographic variables. The diversity in study participants provided a comprehensive understanding of the mental health challenges faced by veterinary students across different contexts. Key demographic factors examined included gender, academic year, age, relationship status, and living arrangements. This broad demographic analysis allowed for the identification of specific subgroups within the veterinary student population that are particularly vulnerable to mental health issues.

### Common stressors

The reviewed studies consistently highlighted several stressors that significantly impact the mental health of veterinary students. The rigorous academic curriculum, high expectations, and demanding clinical training create an environment of intense pressure and constant stress. For example, Strand et al. reported elevated levels of time pressure, depression, and perceived stress among veterinary students (8). Similarly, Hafen Jr. et al. (9) and Hafen et al. (10) identified homesickness, unclear academic expectations, and worries about academic performance as predictive of anxiety and depression (9, 10). The demanding nature of veterinary education, coupled with the emotional burden of caring for patients and dealing with clients, exacerbates these stressors. Additionally, the competitive atmosphere and fear of academic failure further contribute to the high levels of stress experienced by veterinary students (10).

### Gender differences

Gender differences were prominent, with women consistently reporting higher levels of stress, anxiety, and depression compared to men. Studies by Strand et al., Reisbig et al., and Hafen Jr. et al. found that women exhibited greater perceived stress, lower life satisfaction, and poorer general health (8, 9, 11). Karaffa et al. observed that women were more likely to engage in non-suicidal self-injury and reported higher anxiety levels (12). These findings suggest that veterinary students who are women may be more vulnerable to mental health issues, possibly due to societal pressures, gender-specific stressors, and a higher tendency to internalize stress. The need for gender-specific interventions and support systems to address these disparities is evident.

### Academic year and mental health

Mental health issues also varied across different academic years, with certain stages of veterinary education being particularly challenging. Strand et al. (8) described a statistically significant difference amongst class years in terms of perceived stress. Older students exhibited a better attitude and higher levels of satisfaction as well as healthier coping strategies, likely contributing to lower levels of perceived stress. One contributing factor to higher levels of stress during the initial years could be homesickness, as Hafen Jr. et al. (9) found homesickness to be a significant predictor of depression amongst first-year students. Other studies, Siquiera et al. (5) and Hafen et al. (10) also underscored the role of homesickness in students’ mental health. Miller et al. found that self-esteem was lowest among second-year students, while Drake et al. reported that second-year students had the highest levels of psychological distress (13, 14). This indicates that the second year of veterinary school may be a critical period for mental health intervention. The transition from preclinical to clinical training, increased academic workload, and higher expectations during this year may contribute to heightened stress levels. In contrast, third and fourth-year students might have developed better coping mechanisms, explaining their relatively lower distress levels (13, 14).

### Social and relationship factors

Social and relationship factors played a crucial role in the mental health of veterinary students. Hafen et al. and Britt-Lutter et al. found that students in low-satisfaction romantic relationships reported higher depressive symptoms and poorer physical health (15, 16). Additionally, social support, particularly living arrangements, significantly mitigated stress. Chigerwe et al. reported that students living with other veterinary students had lower burnout scores (17). These findings underscore the importance of social interactions and support systems in maintaining mental health. Students who feel isolated or lack social support are more likely to experience negative mental health outcomes. Encouraging peer support groups, mentoring programs, and fostering a sense of community within veterinary schools could help alleviate some of these issues (15–17).

### Coping mechanisms

Veterinary students employed various coping mechanisms, ranging from healthy strategies like exercise and social activities to potentially harmful behaviors such as substance use. Hofmeister et al. highlighted the use of over-the-counter medications and energy drinks, which were associated with higher levels of stress, anxiety, and depression (18). Conversely, Strand et al. noted that exercise and spending time with family and friends were effective methods for relieving stress (8). The diversity in coping strategies reflects the varying degrees of success students have in managing their stress. While some adopt positive coping mechanisms, others may resort to harmful behaviors that exacerbate their mental health issues. Developing programs that promote healthy coping strategies and provide education on the risks of substance use could significantly benefit veterinary students (8, 18).

### Utilization of mental health services

A significant proportion of veterinary students used mental health services, though barriers to access remained. Karaffa et al. found that many students with elevated depression and anxiety scores had used mental health services, but barriers still prevented some students from seeking help (12). Female students were more likely to use mental health services compared to their male counterparts. Common barriers included stigma associated with mental health issues, lack of time, and insufficient awareness of available resources. Addressing these barriers by promoting mental health awareness, reducing stigma, and ensuring accessible mental health services is crucial. Schools could implement mandatory mental health training and provide easily accessible counseling services to support students (12). It is important to acknowledge budgeting requirements for the school to receive this kind of service to provide this kind of service. Furthermore, this can be a dedicated psychologist that the whole university shares.

### Self-esteem and empathy levels

Self-esteem and empathy levels also influenced mental health outcomes among veterinary students. Miller et al. reported that self-esteem varied by academic year, with second-year students having the lowest scores (13). Schoenfeld-Tacher et al. found that empathy levels decreased over time in veterinary students, with first-year students showing higher perspective-taking scores compared to later years (19). This decline in empathy could be due to the emotional exhaustion and stress associated with the demanding veterinary curriculum. Maintaining high levels of empathy is essential for veterinary practice, as it improves client communication and animal care. Interventions aimed at preserving empathy and boosting self-esteem throughout the veterinary education journey are necessary (13, 19).

### Burnout and substance use

Burnout and substance use were significant issues among veterinary students. Chigerwe et al. highlighted that living arrangements affected burnout levels (17). The high prevalence of burnout reflects the intense demands placed on veterinary students, including long hours, high workload, and emotional strain from clinical duties. Addressing burnout through wellness programs, workload management, and promoting work-life balance is critical for the wellbeing of veterinary students. Substance use interventions and education about the risks associated with energy drinks and medications should also be prioritized (17, 18).

### Physical health and academic concerns

Poor physical health and concerns about academic performance were strongly correlated with mental health issues among veterinary students. Physical health is defined as the option functioning of the body in terms of its ability to perform daily activities as well as its contributions to wellbeing. Siqueira et al. and Hafen et al. found that students with poorer physical health and greater academic concerns were more likely to report higher levels of depression and anxiety (10, 20). Physical health issues, often resulting from the sedentary lifestyle and long hours associated with veterinary education, exacerbate mental health problems. Promoting physical health through regular exercise, healthy eating habits, and sufficient rest can positively impact students’ mental wellbeing. Strand et al. (8) found that the most highly reported method of relieving stress amongst veterinary students was exercise. Physical activity has been shown to reduce rates depression and anxiety, and boost overall mood and cognitive function, thereby also impacting academic performance. Addressing academic concerns through effective academic support, clear communication of expectations, and providing resources for academic success is equally important (10, 20).

### Summary

The findings from the reviewed studies reveal the vast range of mental health challenges faced by veterinary students. High academic demands, gender differences, social and relationship factors, common stressors, coping mechanisms, and the utilization of mental health services all play significant roles in shaping the mental health landscape for veterinary students. Addressing these issues through comprehensive support systems, targeted interventions, and effective coping strategies is crucial for improving the overall wellbeing of veterinary students. Schools and educators must prioritize mental health by fostering a supportive environment, providing necessary resources, and implementing proactive measures to mitigate stress and promote resilience (10, 12).

## Discussion

The findings from this review highlight the complex nature of mental health challenges faced by veterinary students. Stress, anxiety, and depression among this population is a significant concern, especially when compared to the general population (21–23). The rigorous academic and clinical demands of veterinary education, coupled with unique stressors such as the emotional burden of animal care and interactions with pet owners, create a high-pressure environment that adversely affects students’ mental health (1).

One of the most striking findings is the pronounced gender differences in mental health outcomes, with women consistently reporting higher levels of stress, anxiety, and depression than men in the reviewed literature (24, 25). This suggests that students who are women may experience additional pressures or have different coping mechanisms when compared to their that need to be addressed through gender-specific interventions. For example, women were significantly more inclined than men to employ coping strategies such as acceptance, self-distraction, positive reframing, and seeking emotional support. Notable interactions between gender and coping strategies were also observed (26). The higher prevalence of self-harm (non-suicidal) and anxiety among women further shows the need for targeted support and resources to help them manage their mental health more effectively (27).

The variation in mental health issues across different academic years indicates that certain stages of veterinary education are more challenging compared to other points in the curriculum. The second year of student life in veterinarian school appears to be a critical period where students experience the lowest levels of self-esteem and the highest levels of psychological distress (14). This phase often involves the transition from preclinical to clinical training, increased academic workload, and heightened expectations, all of which contribute to significant stress. Interventions customized to the needs of students at this stage via mentorship programs and stress management workshops could help to counteract these challenges (28).

Social support and relationship factors play a crucial role in the mental health of veterinary students (29). Homesickness appears to play a critical role in the mental-health of first year students. The findings in regard to students in romantic relationships that leave more to be desired and those with insufficient social support, highlight the importance of fostering strong social networks as these students show higher levels of depression when compared to their peers (1, 10). Encouraging peer support groups, facilitating social interactions, and promoting a sense of community within veterinary schools can help alleviate feelings of isolation and provide a buffer against stress (12).

The diversity in coping mechanisms employed by veterinary students reflects varying degrees of success in managing stress. While healthy strategies like exercise and social activities are beneficial, the use of potentially harmful behaviors such as substance use is concerning (30). Additionally, although a significant proportion of students utilize mental health services, barriers to access, such as stigma and lack of awareness, remain. Developing programs that promote healthy coping strategies and provide education on the risks of substance use is essential. Systems level strategies such as campus groups that raise awareness about mental health and substance use issues are particularly effective on university campuses. Furthermore, peer support training to provide peer-based therapy on campus, as well as mental health workshops around stress management and mindfulness can be effective campus wide strategies to promote wellbeing (31). Efforts to reduce stigma, increase awareness of available resources, and ensure easy access to mental health services are crucial steps in supporting students’ mental wellbeing (32).

The decline in self-esteem and empathy levels over the course of veterinary education is alarming. Empathy, the underlying tone of successful veterinary care, appears to dwindle as student’s progress through their schooling. This decline could be due to emotional exhaustion and the cumulative stress of the demanding curriculum. Interventions aimed at preserving empathy and boosting self-esteem should be investigated, such as empathy training and wellness programs. Furthermore, the high prevalence of burnout among veterinary students highlights the need for institutional changes to manage workload and promote work-life balance (19, 33). For example, sessions could be held to increase the level of stress relief in students, such as holding a painting day, or a yoga day where classmates can get together and unwind.

Resources for physical health as well as academic support play a critical role in improving student mental health. The strong correlation between poor physical health and mental health issues underscores the importance of promoting physical health among veterinary students. Encouraging regular exercise, healthy eating habits, and sufficient rest can positively impact students’ mental health (34). Additionally, providing subsidized or free-of-cost access to a gym membership or exercise classes can be a good way to reduce the barrier of entry to physical activity. Addressing academic concerns through effective academic support, clear communication of expectations, and providing resources for academic success is equally important. Key interventions for academic include institution based support such as an academic resource center and study groups, with additional individualized supports for students who need accommodations or are struggling in terms of their performance. These measures can help reduce the stress associated with academic performance and improve overall wellbeing (35).

Future research should focus on longitudinal studies to track the mental health of veterinary students throughout their education and into their professional careers. Understanding the long-term impact of veterinary education on mental health can help identify critical periods for intervention and inform the development of effective support programs. Additionally, exploring the effectiveness of specific interventions, such as mindfulness training, stress management workshops, and peer support groups, can provide evidence-based strategies for improving mental health outcomes (1). Finally, additional research on this matter should be focused on the role of cultural and institutional factors in shaping the mental health of veterinary students. By doing this, a more personalized intervention that addresses the unique needs of diverse student populations will emerge.

## Limitations

The findings of this review should be interpreted within the context of the following limitations. The review was limited to the databases selected for review, which may have led to the researchers inadvertently excluding published research from the review. However, the researchers conducted a comprehensive search, including the Google Scholar database, with an intention to identify all relevant studies for inclusion. Additionally, the search was limited to studies published in English, which may have prevented inclusion of studies published in other languages. The focus on mental health as a research outcome was also a limitation, as there was variation in the operationalization of mental health in the reviewed studies (i.e., stress, coping, self-esteem, depression, empathy). Lastly, the review includes a synthesis of the research findings from the identified studies rather than a meta-analysis, as this was not the intention of this research.

## Conclusion

The review of the literature reveals the wide variety of mental health challenges faced by veterinary students. High academic demands, gender differences, social and relationship factors, common stressors, coping mechanisms, and the utilization of mental health services all significantly impact mental health for these students. Addressing these issues through well-built support systems, targeted interventions, and effective coping strategies is essential for not only improving the overall wellbeing of veterinary students, but their stress levels as well. Veterinary schools should do their best to prioritize their students’ mental health level by fostering a supportive environment, providing necessary resources, and implementing proactive measures to mitigate stress and promote resilience. By doing so, this will ensure that future veterinarians are well-equipped to manage the demands of their profession while maintaining their mental health and wellbeing.
