# Supplementary material for: Exploring mental health in veterinary students: common stressors and effective coping strategies: a narrative review
Source: Front Vet Sci. 2025 Feb 11;12:1470022. doi: 10.3389/fvets.2025.1470022 (PMC11866424; doi:10.3389/fvets.2025.1470022)
Supplement: Supplementary file 1 [file Table_1.docx]

Supplementary Table 1. Table of Studies that Address Factors in Assessment of Mental Health in Veterinary Students.

| Author and Year | Sample | Correlates & Factors | Measurement of Mental Health | Significant Findings & Associations |
| --- | --- | --- | --- | --- |
| Strand et al., 2005 | Veterinary students, *n*=157, 80% female, mean age=27 years | Demographic characteristics including age, gender, living companions, animal companions, marital status, academic classification, and % cash flow coming from loans | Assessment of students’ current experiences of perceived stress and its relationship to various demographic characteristics | Relative to the general population, veterinary students experience higher levels of time pressure (M=55.51, SD=9.23, *P*<0.001), higher levels of depression (M=53.37, SD=11.02, *P*<0.001), and higher levels of perceived stress (M=55.37, SD=10.49, *P*<0.001)  Statistically significant differences were found between academic years and genders on subjective accounts of perceived stress *t* scores (*F*(3,149)=5.16, *P*=0.002)  Females across academic classes indicated higher levels of perceived stress (M=56.90, SD=10.19) when compared to their male counterparts (M=48.83, SD=9.33; *F*(1,150)=16.00, *P*<0.001)  There was a positive relationship between perceived stress and the number of animal companions (*P=*0.029)  For total stress scores, females scored higher (M=48.94, SD=10.50) than males (M=43.00, SD=7.32); *P*=0.003  There were statistically significant differences across academic classes on attitude posture (*F*(3,152)=3.37, *P*=0.020), vocational satisfaction (*F*(3,152)=8.29, *P*<0.000), and health posture (*F*(3,152)=3.48, *P=*0.018)  Positive correlations were found among the number of animal companions and time pressure (*r_s_*=0.225, *P*=0.005), driven behavior (*r_s_*=0.216, *P*=0.007), role definition (*r*_s_=0.272, P=0.001), anxiety (*r_s_*=0.220, *P=*0.006) and depression (*r_s_*=0.269, *P=*0.001)  The older students were, the more likely they were to report desirable healthy attitudes and behaviors (*r_s_*=-0.181, *P*=0.025)  Best method of relieving stress (% response):  Exercise (47%)  Spending time with family/friends (26%)  Hobbies (22%) |
| Hafen et al., 2006 | First-year veterinary students, *n*=93, 71% female, mean age=24 (SD=2.9, range=21-29) years, 88% Caucasian  Relationship status:  Dating (36%)  Single (35%)  Married (18%)  Cohabitating (11%) | Demographic characteristics including age, gender, relationship status as well as students’ reported stressors | The extent to which students’ age, gender, and specific reported stressors were predictive of anxiety or depression | Predictive stressors were homesickness, unclear instructor expectations, and worries about not being as smart as other students. This model explained 47% of the variance for veterinary medical student depression (*F*=11.97, *P*<0.001)  For anxiety, results showed significant predictors of perceived physical health and 3 specific stressors (homesickness, unclear instructor expectations, and being behind in studies); this model accounted for 44% of the variance of anxiety within veterinary students (*F*=9.16, *P*<0.001) |
| Hafen et al., 2008 | First-year veterinary students, *n*=78, 77% female, mean age=24 (range=21-34, SD=2.6) years, 91% Caucasian  Relationship status:  Dating (36%)  Single (34%)  Married (21%)  Cohabitating (9%) | Demographic characteristics including age, gender, ethnicity, and relationship status, as well as students’ reported stressors during the first year of veterinary school (first and second semester) | The extent to which students’ specific reported stressors correlated with depressive symptoms (reflected by their classification as “adaptive” versus “struggling”) | During first semester (mean=14.31, SD=9.26) and second semester (mean=13.30, SD=7.63) students reported elevated depression levels; 32% of first-semester students and 28% of second-semester students scored above the clinical cut-off for depression  Those with concerns about academic performance originating during the first semester were 6 times as likely to be struggling in coping with depressive symptoms ($\beta$=1.80, OR=6.05; *P*<0.05)  When struggling is predicted using only first-semester stressors, those students reporting homesickness ($\beta$=1.03, OR=2.80) or concerns about academic performance (𝛽=1.00, OR=2.72) are nearly three times as likely to be categorized as struggling than those without these concerns (*P*<0.05)  Second-semester respondents self-reporting poorer physical health were more than five times as likely as their adaptive counterparts to be categorized as struggling (𝛽=-1.66, OR=0.19; *P*<0.05)  Students who express concerns about fitting in with peers during the second semester are nearly four times as likely as those without similar concerns to be considered struggling (𝛽=1.36, OR=3.91; *P*<0.05) |
| Hofmeister et al., 2010 | Veterinary students, *n=*456, median age=24 (range=19-50) years, 72% female | Demographic characteristics including age, gender, year in school, species focus in school, race, marital status, body weight, BMI, and GPA  Consumption/use of OTCs and Energy Drinks (ED), daily caffeine intake, health status, perceived level of rest and exhaustion were also inventoried | Use of OTC medications (for stimulant, depressant, or nootropic effects) and how their use by veterinary students relates to measures of anxiety, stress, and depression | Use of OTC or ED product in the past month (UGA vs. CSU %):  None (52% vs. 46%)  ED (30% vs. 30%)  Energy booster (15% vs. 12%)  Something for fatigue (20% vs. 18%)  Antihistamine (22% vs. 15%)  Kava (0% vs. 1%)  St. John’s wort (1% vs. 1%)  Melatonin (7% vs. 2%)  Pseudoephedrine (12% vs. 8%)  Valerian root (2% vs. 1%)  A nootropic (4% vs. 4%)  Smoking (9% vs. 15%)  Alcohol (80% vs. 85%)  Illicit drugs (4% vs. 12%)  Reason for using OTC stimulant (UGA vs. CSU %)  Aid studying (63% vs. 71%)  Wakefulness during the day (60% vs. 60%)  Waking up in the morning (52% vs. 62%)  Aid driving for a long period (32% vs. 35%)  Get more energy during the day (30% vs. 50%)  Improve mood (13% vs. 24%)  Mix with alcohol (7% vs. 5%)  Stress coping (6% vs. 6%)  Treat a hangover (6% vs. 4%)  Aid weight loss (3% vs. 1%)  Manage anxiety (2% vs. 4%)  Reason for using OTC depressant (UGA vs. CSU %)  Fall asleep at night (48% vs. 55%)  Help stay asleep at night (30% vs. 48%)  Stress coping (7% vs. 5%)  Manage anxiety (5% vs. 18%)  Improve mood (5% vs. --)  Prevent or treat depression (4% vs. 3%)  Reason for using OTC nootropic:  (UGA vs. CSU %)  Improve memory (21% vs. 50%)  General health benefits (14% vs. 36%)  Improve learning (7% vs. 43)  Help study (7% vs. 21%) Stress coping (7% vs. 7%)  Improve mood (7% vs. 7%)  Manage anxiety (3% vs. --)  UGA:  OTC medication users were less likely to report feeling exhausted once a week or less (9% vs. 24%; P<0.02) and to be single (68% vs. 76%; P<0.02), and they had higher stress scores (14.7 vs. 11.9; P<0.008) than non-OTC users  Regular OTC medication users were more likely to be classified in the highest caffeine consumption category than non-regular OTC medication users (38% vs. 18%; P<0.04)  UGA: 43% were Energy drink users, of those, 47% are regular ED users  ED users were:  Likelier to be classified in the highest caffeine consumption category (31% vs. 15%; P<0.04)  Likelier to be equine or large-animal focused (25% vs. 13%; P<0.02)  Had higher caffeine consumption values after controlling for the contribution of ED caffeine (297 vs. 205mg; P<0.04)  Reported fewer hours of sleep (5.9 vs. 6.3; P<0.02)  Higher anxiety scores than non-ED users (6.4 vs. 5.2; P<0.05)  Regular ED users were:  Likelier to report never feeling rested (30% vs. 8%; P<0.03)  Likelier to report feeling exhausted once or more a day (43% vs. 25%; P<0.04)  Likelier to be classified in the highest caffeine consumption category (39% vs. 24%; P<0.04)  Likelier to be female (82% vs. 64%; P<0.05)  Had higher stress scores than non-regular ED users (16.4 vs. 11.0; P<0.008)  Compared to OTC users, ED users were:  Likelier to be single (77% vs. 57%; P<0.03)  Consumed more caffeine (320 vs. 213mg; P<0.05)  CSU: 44% were Energy Drink users, of those, 45% were regular ED users  Compared to non-ED users, ED users reported higher anxiety scores (3.2 vs. 2.0; P<0.006) and were:  Likelier to be male (26% vs. 15%; P< 0.04)  Likelier be single (82% vs. 65%; P<0.02)  Likelier to report feeling exhausted more than once a day (32% vs. 19%; P<0.01)  Less likely to report feeling tired in class once a week or less (11% vs. 27%; P< 0.02)  Compared to non-regular ED users, regular ED users:  Were likelier to be placed in a higher caffeine consumption group (37% vs. 25%; P<0.05)  Were likelier to be on prescription medication (16% vs. 4%; P<0.04)  Used more caffeine per day (387 mg vs. 270 mg; P<0.03)  Had higher depression scores (5.4 vs. 2.4; P<0.002)  Had higher anxiety scores (4.2 vs. 2.4; P<0.005)  Had higher stress scores (7.9 vs. 5.5; P<0.05)  Compared to OTC users, ED users were less likely to report feeling tired in class once a week or less (10% vs. 33%; *P<*0.004)  UGA juniors reported higher scores for depression than freshmen (9.5 vs. 6.7, *P<0*.05)  CSU juniors reported higher depression, anxiety, and stress scores than did freshmen (5.1 vs. 2.5, 3.3 vs. 2.1, and 8.0 vs. 5.1, respectively; all *Ps*<0.003)  CSU sophomores reported higher depression scores than freshmen (4.2 vs. 2.5; *P*<0.0001)  CSU students reported significantly lower depression (3.5 vs. 7.9), anxiety (2.5 vs. 5.7), and stress (6.0 vs. 12.9) scores than did UGA students (all *Ps*<0.0001) |
| Reisbig et al., 2012 | Veterinary students, *n*=304, female (79% for first-semester participants, 79% for second-semester participants, and 81% for third-semester participants); 1^st^ semester participants’ mean age=23.6, (range=21-36, median=23) years, 2^nd^ semester participants’ mean age=25.1 (range=22-42, median=23) years, and 3^rd^ semester participants’ mean age=24.6 (range=23-35, median=24)  Race/ethnicity:  Caucasian (96%)  Asian (1%)  Hispanic/Latino (2%)  Other (1%)  Relationship status:  Not in a relationship (33%)  Dating (30%)  Married (18%)  Engaged (9%)  Living together (6%)  Long distance (1%)  Divorced/annulled (<1%)  Domestic partner (<1%)  Other (1%) | Demographic variables including age, relationship status, race/ethnicity, religious belief  Inventory of stressors, anxiety symptoms, depressive symptoms, plus measures for overall life satisfaction, perception of general health, perception of academic performance, and self-reported GPA for the previous semester | The relationship between common stressors experienced by veterinary students and factors predictive of poor outcomes in areas of mental health, general health, and academic performance | For first-semester general health, gender negatively predicted and predicted for 5% of the variance (*P*<0.001); the four stressors (academic, transitional, family-health, relationship) accounted for an additional 13% of the variance in which academic stress (*P*<0.001) and transitional stress (*P*<0.05) negatively predicted first-semester general health  Among 1st-semester measures, female students reported higher levels of anxiety (*P*=0.002) and academic stress (*P*=0.002) and lower levels of life satisfaction (*P*=0.002) and general health (*P*=0.001) compared to male students at *P*<0.006  Female students’ depression scores during all 3 semesters were higher than the clinical cut- off point (1st semester *t*[202]=3.94; 2nd *t*[114]=3.98; 3rd *t*[153]=6.64; all *P*s<0.001)  Male students’ first semester depression scores were lower than the clinical cut-off point (*t*[51]=-2.79; *P*<0.01)  The overall depression symptom levels increased across the three semesters and impacted a large percentage of the students each semester (% scoring at or above clinical cut-off male vs. female)  1st semester: 30% vs. 54%  2nd semester: 52% vs. 68%  3rd semester: 53% vs. 73%  Female students experienced a higher level of depression than male students during the first and third semesters; they also experienced a higher level of anxiety than male students during all 3 semesters (*Ps*<0.001)  Females experienced a lower level of life satisfaction than male students during the 1^st^ and 3^rd^ semesters (*P*<0.01); they additionally reported a lower level of general health than male students during the 1st and 2nd semesters (*P*<0.01)  Students who reported a high level of academic stress and transitional stress were more likely to report a high level of depression and anxiety (*P*<0.001); additionally, academic stress (*P*<0.001) and transitional stress (*P*<0.05) negatively predicted first-semester life satisfaction  Those who experienced a high level of relationship stress during the first semester were likely to report a high level of anxiety during that semester (*P*<0.01)  Religious belief positively predicted and accounted for 3% of the variance in academic performance (P<0.05); the four stressors (academic, transitional, family-health, and relationship) accounted for an additional 22% of the variance (P<0.001) in academic performance  Students who reported a high level of academic stress during the second and third semesters were more likely to report a low GPA for the second and third semesters (P<0.05); those experiencing high family-health stress during the third semester were more likely to report a low GPA during that semester (*P*<0.05) |
| Siqueira et al., 2012 | Veterinary students, *n*=142, 73% female, 96% Caucasian, mean age=23.7 years | Demographic variables including age, gender, race/ethnicity  Inventories were used to assess perceived stressors, symptoms of anxiety, and symptoms of depression | Determine the extent to which demographic variables and specific stressors predict anxiety and depression | Mean anxiety and depression scores spike during second (anxiety mean=17.65, depression mean=19.47) and third (anxiety mean=17.45, depression mean=19.67) years of veterinary school  For depression, perceived physical health, difficulty fitting in with classmates, unclear expectations of professors, heavy workload, and homesickness were predictive of elevated depression scores; this model explained 64% of the variance in veterinary students’ depression scores (*F*=48.85; *P*<0.001)  Those who perceived faculty to have unclear expectations, had more difficulty fitting in, perceived their workload as heavy, and perceived to have poor physical health were more likely to have high anxiety scores (*P*<0.001) |
| Hafen et al., 2013 | Veterinary students, *n=*240, 72% female, 94% Caucasian, mean age=24 (SD=2.6, range=21-35) years  Relationship status:  Married (27%)  Non-marital romantic relationship (48%)  Not in a relationship (25%) | Demographic variables including age, gender, race/ethnicity, relationship status  Other measures include relationship satisfaction, depressive symptoms, anxiety symptoms, perceived stressors, and perceived health | The extent to which relationship satisfaction influences overall well-being as measured by perceived health, school/life balance, perception of unclear expectations, and depression | Students involved in low satisfaction romantic relationships were likelier to experience elevated depressive symptoms (*t=*3.33; *P*<0.01), poorer physical health (*t*=-2.43; *P*<0.05), more challenges in maintaining a balance between school and personal life (*t*=3.61; *P*<0.01), and more difficulty coping with academic expectations (*t=*1.99; *P*<0.05)  Relationship satisfaction is negatively associated with both elevated relationship conflict (*P*<0.001) and heightened stress surrounding balancing school and personal life (*P*<0.05)  Compared to those not in a relationship, those in a relationship reported greater stress with family illness (*t*=-2.13; *P*<0.05), relationship conflict (*t*=-4.29; *P*<0.01), and school/life balance (*t*=-10.00; *P*<0.01)  Students reporting higher relationship satisfaction were more likely to experience elevated stress from being behind in their studies (*P*<0.05) |
| Chigerwe et al., 2014 | Veterinary students, *n*=293 | Demographic variables including age, gender, # dependents in household, marital status, and living arrangement (with spouse vs. with another vet student vs. with a non-vet student vs. alone) | Evaluation of the Maslach Burnout Inventory as a tool to assess burnout and evaluation of factors that predict MBI-ES scores (including subscale measures of emotional exhaustion, depersonalization, and low personal accomplishment) | Scores for emotional exhaustion were higher in the spring compared to scores in the fall semester (*P*=0.0002)  Living arrangements, specifically whether a student lived with another veterinary medical students or not was the only independent variable significantly associated with the MBI-ES scores in this group of students; veterinary students living with another student were more likely to have scores indicating low degree of burnout (*P*=0.031) |
| Diulio et al., 2015 | Veterinary students, *n*=210, 79% female, 95% Caucasian, mean age=24.5 (range=19-43, SD=3.3) years  Academic classification:  1st year (59%)  2nd year (22%)  3rd year (12%)  4^th^ year (7%)  Relationship status:  Single (69%)  Living with a partner (10%)  Married (18%)  Separated (1%)  Divorced (1%)  Widowed (1%)  Unspecified (<1%) | Demographic variables including gender, race/ethnicity, age, academic classification, and relationship status  Presence and severity of depressive symptoms, severity of emotional distress, drinking behaviors, and drinking motives | Severity of emotional distress and presence of depression as measured by the Center for Epidemiological Studies Depression Scale Short Form (CES-D-SF)  The nature of alcohol consumption and behaviors was assessed using the Drinking Motive Questionnaire-Revised (DMQ-R); in addition, the Alcohol Use Disorders Identification Test (AUDIT) was used to assess harmful alcohol consumption patterns  The Daily Drinking Questionnaire (DDQ) was used for participants to record current drinking patterns, including average and maximum alcohol consumption per day of the week and number of binge episodes in the past 28 days | Compared to women, men reported more frequent binge episodes (*P*<0.01), greater AUDIT scores indicating increased likelihood of alcohol-related harm (*P*<0.01), and higher weekly consumption of alcohol (*P*<0.01)  The relationship between symptoms of depression and AUDIT scores was mediated by coping motives (*P*<0.001)  Drinking to manage internal states and rewards (coping, *P*=0.04; enhancement motives, *P*<0.001) was associated with risky drinking among veterinary students  The majority of participants reported experiencing at least one symptom of depression during the past week (95.5%), and, on average, participants reported experiencing about four (M=3.8) different symptoms of depression during that time period |
| Miller et al., 2015 | Veterinary students, *n=*1653, 81.2% female, 44.4% between the age of 23-25 years  Academic classification:  Incoming students (24%)  First year (22%)  Second year (23%) Third year (18%)  Fourth year (13%) | Demographic information was collected including age, gender, academic classification, tuition status (in vs. out of state), degrees held before entry to veterinary school  Measures of self-esteem, current-year experience, perception of the learning environment, perceived stress, and perceived skills were also taken | Evaluation of student self-esteem and its relationship to gender, age, as well as measures of stress, tuition status, GPA, learning environment, technical and non-technical experiences, and skill development over the course of a 4-year curriculum | The standardized self-esteem (SE) score was lowest in Year 2 responses (70.88, 95% CI [69.13–72.64]) and highest among responses at orientation (86.0, 95% CI [84.8–87.2]) and responses from Year 4 (83.3, 95% CI [81.28–85.31], 𝝌^2^=189.4, *P*<0.0001)  43% of high SE responses were from students over 25 years of age compared to only 35% of low SE responses (𝝌^2^=32.12, *P*<0.001)  Regarding gender, 22% of high SE responses were from males, compared to 16% males among the low SE responses (𝝌^2^=8.95, *P*=0.003)  High SE group responses had higher cumulative GPAs (𝝌^2^=37.75, *P*<0.001) and previous semester GPAs (𝝌^2^=69.36, *P*<0.001) compared to low SE responses  Three stress indices (isolation, academic, competition) were higher in the lower SE responses, while all other indices (learning environment, experiences, skill development) were higher in high SE responses (all *Ps*<0.0001) |
| Schoenfeld-Tacher et al., 2015 | Veterinary students, *n*=361, 77% female  CSU mean age=25 (median=24) years  NCSU mean age=24 (median=23) years | Demographic information was gathered including age, gender, and degree held at the time of matriculation  Self-reported measures of empathy including cognitive and affective components were also inventoried | Measures of empathy (cognitive and affective) were taken using the Interpersonal Reactivity Index (IRI) which assesses empathetic concern (EC), personal distress (PD) and perspective taking (PT)  PT scores are positively correlated with better social functioning and higher self-esteem  PD is a measure of participants’ self-oriented feelings of anxiety and unease in interpersonal settings; higher scores in this domain are associated with higher levels of social dysfunction and lower levels of social competence  The EC scale assesses ‘‘other-oriented’’ feelings of sympathy and concern for unfortunate others | Over time, Perspective Taking (PT) (*F*=7.698, *P=*0.001) scores decreased, while mean Personal Distress (PD) scores (*F*=4.249, *P*=0.015) increased  PT scores were significantly higher for first-year students (T1 mean=20.29, SD=3.90) than either of the other two groups (T2 mean=18.43, SD=4.73; T3 mean=18.65, SD=4.20) showing a sharp decline early in veterinary students’ education between T1 and T2 |
| Drake et al., 2017 | Veterinary students, *n*=279, 80% female, mean age=26 years (median=26, SD=4.2) years  Race/ethnicity:  Caucasian (87.2%)  Asian (5%)  Hispanic (3.5%)  African American (1.8%)  Native American (1%)  Academic classification:  1^st^ year (46%)  2^nd^ year (24.7%)  3^rd^ year (18.6%)  4^th^ year (10.4%)  Relationship status:  Single (71.4%)  Married (18.4%)  Cohabitating with partner (4.2%)  Religious affiliation:  None (31.9%)  Catholic (22.5%)  Protestant (21%)  Other (20%) | Demographic characteristics including gender, age, race/ethnicity, academic classification, relationship status, and religious affiliation were recorded  Other self-report measures were taken including an inquiry of physical health and presence/severity of psychological distress | Examination of the demographic variables of help-seeking veterinary students; the rate at which support is sought, perceived physical health, and the level of distress experienced by those students seeking counseling services  In addition to demographic data, students completed the Outcome Questionnaire-45 (OQ-45) a self-report scale which estimates and monitors the degree of psychological distress; it comprises a total score plus three subscales (symptom distress, interpersonal relations, and social role)  Total score indicates overall life satisfaction and well-being  Symptom distress subscale measures overall current emotional functioning (assessing for symptoms of anxiety, depression, and substance use)  The interpersonal relations subscale measures distress in personal relationships  The social role subscale assesses feelings of inadequacy, dissatisfaction, conflict, or distress regarding issues of employment, family roles, or leisure | Slightly over half of students seeking counseling in the study period had total OQ-45 scores above the clinical cut-off (54%)  Students who perceived themselves to be in poor physical health were also more distressed than their counterparts who perceived themselves to be in good physical health (*P*<0.001)  Students in each school year reported scores above the clinical cut-off scores, with second-year students reporting the highest average scores overall and on the symptom distress and social role subscales  Overall score:  1^st^ year: M=64.15, SD=22.92  2^nd^ year: M=69.19, SD=20.87  3^rd^ year: M=64.77, SD=20.83  4^th^ year: M=64.15, SD=24.20  Symptom distress:  1^st^ year: M=38.06, SD=14.38  2^nd^ year: M=40.91, SD=13.51  3^rd^ year: M=37.74, SD=13.44  4^th^ year: M=37.74, SD=16.83  Social role:  1^st^ year: M=13.28, SD=4.52  2^nd^ year: M=15.12, SD=3.96  3^rd^ year: M=14.02, SD=4.85  4^th^ year: M=12.81, SD=5.08 |
| Kustritz, et al., 2017 | Second-year veterinary students, *n*=57, 89.5% female, mean age=24.7 (SD=2.5) years | Demographic characteristics including gender, age, and GPA from the previous school year were collected  Additional data were collected including a mindset survey and perceived stress scale | Demographic characteristics, in addition to measures of mindset, perceived stress, and life change were collected to determine whether there is a correlation between mindset and GPA or between mindset and self-assessed stress levels | Overall mean stress score was 16.1±6.5; there was no significant association between mindset group and either stress score or first-year GPA |
| Britt-Lutter, et al., 2019 | Veterinary students, *n*=230, 87% female, mean age=24.57 (range=21-43, SD=2.75), 93% Caucasian  Academic classification:  1^st^ year (22%)  2^nd^ year (30%)  3^rd^ year (23%)  4^th^ year (20%) | Demographic characteristics including gender, age, race/ethnicity, academic classification, current student loan debt, and credit card debt were collected  Other information was assessed including perceived intelligence, small animal focus, expected income after completion of veterinary school, anticipated student loan debt, relational satisfaction, financial satisfaction, and depressive symptoms | Demographic characteristics, measures of mental health status, and life satisfaction (relational and financial) were variables collected in order to determine the extent to which these variables correlate with depressive symptoms  Mental health status was captured using the Patient Health Questionnaire (PHQ-9) | 26% of veterinary students met the initial screening cut-off for moderate or above depressive symptoms; 37% met criteria for having mild depression  Vet students with lower relational satisfaction (*β*=−0.211, *P*=0.002) and financial satisfaction (*β*=−0.192, *P*=0.003) were more likely to report depressive symptoms  Current student loans and expected student loans had a negative effect on financial satisfaction (*β*= −0.194, *P*<0.001 and *β*=−0.321, *P*<0.001, respectively)  Compared to first year students, third year students were more likely to report depressive symptoms (*β*=0.179, *P=*0.037) |
| Karaffa et al., 2019 | Veterinary students, *n*=573, 86.9% female, median age=25 (range=21-50) years, 94.4% Caucasian, 83.4% heterosexual  Relationship status:  Married (17%)  Single (>35%)  Dating/committed relationship (≈46%)  Household/domestic status:  Have children (5.6%)  Living with roommates (44.7%)  Living with a partner or spouse (31.2%) | Demographic information included age, gender identity, race/ethnicity, sexual orientation, relationship status, living situation, parent status, academic classification, and current GPA  Data were collected regarding presence/severity of depressive symptoms, presence/severity of anxiety symptoms, alcohol use behaviors, self-injury and suicidality, and prior use of mental health services | Measures of mental health were taken using various instruments to assess for depression (Patient Health Questionnaire; PHQ-9), anxiety (Generalized Anxiety Disorder 7-Item; GAD-7), alcohol use disorder (Alcohol Use Disorders Identification Test-Consumption; AUDIT-C), self-harm or suicidality (Non-Suicidal Self Injury; NSSI) to determine prevalence rates of these disorders and their relationship(s) to gender or other variables  Use of prior mental health services was also assessed to determine the proportion of students who have used these services and what proportion of students with elevated scores on measures of depression, anxiety, or alcohol use have used mental health services | Participants who reported engaging in NSSI were more likely to report having seriously thought about suicide (χ^2^=65.56, *P*<0.01, *φ*=0.38) or having made a suicide attempt (χ^2^=40.20, *P*<0.001, *φ*=0.26)  Depression scores on the PHQ-9 were highly positively correlated with anxiety scores on the GAD-7 (*P*<0.001)  Women reported higher levels of anxiety (mean=8.64, SD=5.56) on the GAD-7 compared to men (mean=6.54, SD=4.94) (*P*<0.01)  Women were more likely to report engaging in NSSI than men (χ^2^=7.97, *P*=0.005, *φ*=−0.12)  69% of participants reported using some form of mental health service in the past and described their experience as:  Very positive (18%)  Positive (35%)  Somewhat positive (22%)  Neutral (11%)  Somewhat negative, Negative, or Very negative (14%)  Timeline of mental health service use:  Currently engaging in some form of mental health service (24%)  Within the past month (>5%)  Within the past year (13%)  Within the past 1-5 years (15%)  More than 5 years ago (12%)  Of the participants who scored at or above a clinical cut-off of 10 on the PHQ-9 (33.9%), corresponding with moderate or higher depression severity, ≈79% reported having a history of using mental health services  Of the participants who scored at or above a clinical cut-off score of 10 on the GAD-7 (36.2%), corresponding with moderate or higher anxiety, ≈78% reported using some form of mental health services currently or in the past  Among participants who scored at or above a clinical cut-off of 5 on the AUDIT-C (25%), ≈66% reported having a history of using some form of mental health service |
| Nahar et al., 2019 | Veterinary students, *n*=264, mean age=25.3 (SD=3.21) years, 88.3% female, 89.8% Caucasian/White  Academic Classification:  1^st^ year (33.2%)  2^nd^ year (25.7%)  3^rd^ year (26.0%)  4^th^ year (14.7%) | Demographic characteristics such as gender, age, academic classification, race/ethnicity, housing/children/domestic status, GPA, and employment status were collected  Perceived stress, anxiety, and depression were also measured | Various demographic characteristics and measures of mental health (stress, anxiety, depression) were collected  Perceived stress was assessed using a 10-item scale (PSS)  Anxiety and depression were measured using a four-item questionnaire (PHQ-4) | 52.3% met or exceeded the scale cut-point indicative of a possible generalized anxiety disorder  22.6% of participants screened positive for depression based on the PHQ-4  Stress levels were elevated among this sample with the mean (20.6±6.62) exceeding that of age and gender matched counterparts  Stress level was correlated with total PHQ-4 score (*r*=0.77, *P*<0.001)  Stress level was also associated with sex and GPA (*Ps*<0.01); female students had a higher likelihood of exhibiting anxiety compared to males in the sample (𝝌^2^=7.721, OR=3.06, 95% CI [1.35-6.91], *P*<0.01)  Those identifying as non-White (𝝌^2^=5.56, OR=2.64, 95% CI [1.51-6.05], *P*<0.05) and those living in on-campus housing (𝝌^2^=6.26, OR=0.27, 95% CI [0.09-0.80], *P*<0.05) were more likely to screen positive for depression |
| Holden et al., 2020 | Veterinary students, *n*= 99, 82.7% female  Age in years (%):  18–22 (26.3%)  23–25 (55.6%)  26–28 (11.1%)  29–31 (3.0%)  32–34 (2.0%)  35–39 (2.0%)  Academic classification (%):  1^st^ year (43.4%)  2^nd^ year (21.2%)  3^rd^ year (15.2%)  4^th^ year (17.2%)  DVM PhD student (3.0%) | Demographic characteristics including gender, age, and academic classification  Other measures included assessment of general personality traits, perfectionism, and resilience | After collection of basic demographic characteristics, three additional inventories were used to assess various personality characteristics including the Multidimensional Perfectionism Scale (MPS), Big Five Inventory (BFI), and the Brief Resilience Scale (BRS)  MPS includes subscales of self-oriented perfectionism, others-oriented perfectionism, and socially prescribed perfectionism  The BFI scale includes subscales for assessing levels of extraversion, agreeableness, conscientiousness, neuroticism, and openness  The BRS is a 6-item instrument used to evaluate levels of resilience | Descriptive statistics for each characteristic (mean±SD):  Self-oriented perfectionism (56.70±9.81)  Others-oriented perfectionism (46.37±9.22)  Socially prescribed perfectionism (44.09±10.00)  Openness (36.36±5.43) Conscientiousness (36.15±5.11)  Conscientiousness (transformed) (3.02±0.85)  Extraversion (25.08±7.19)  Agreeableness (36.34±4.87)  Neuroticism (25.80±4.47)  Resilience (21.56±4.47)  The levels of perfectionism of veterinary students are statistically significantly related to their personality factors (*P*<0.001)  Self-oriented perfectionism was correlated to conscientiousness (*r*=.214, *P*<.034) and also related to neuroticism (*r*=.380, *P*<0.001) |
| Chigerwe et al., 2020 | Second year veterinary students, *n*=102, 99% female, age range=20-25 years  Race/ethnicity:  White/Caucasian (80%)  Asian (29%)  Multi-ethnic (23%)  Hispanic, Latino or Spanish origin (6%)  African American/Black (1%)  Relationship status:  Single (81%)  Relationship with spouse/partner (19%) | Demographic variables including age, gender, race/ethnicity, relationship status were collected  Various instruments were used to assess anxiety, burnout, depression, and quality of life | Anxiety, burnout, depression, and quality of life were assessed using the Generalized Anxiety Disorder (GAD-7), Maslach Burnout Inventory (MBI), Patient Health Questionnaire (PHQ-9), and Short Form-8 (SF-8)  GAD-7 was used as a screening tool and severity measures for generalized anxiety disorder  MBI measures individual burnout levels through subscales of emotional exhaustion, depersonalization, and personal accomplishment  PHQ-9 includes a depression module useful for assessing severity of depressive symptoms  SF-8 assesses health related quality of life with established reliability and validity for the health domain scales of general health perception (GH), physical functioning (PF), role limitations due to physical health problems (role physical, RP), bodily pain (BP), energy/fatigue (vitality, VT), social functioning (SF), role limitation due to emotional problems (role emotional, RE), and psychological distress and well-being (mental health, MH) | Anxiety, GAD-7:  Moderate (median score=8; 95% CI=[7,10])  Depression, PHQ-9:  Mild to moderate (median score=8; 95% CI=[6,9])  Burnout, MBI:  Emotional exhaustion (median score=33; 95% CI=[29,35])  Depersonalization (median score=7; 95% CI=[5,9])  Personal accomplishment (median score=30.0; 95% CI=[28,32])  Quality of life, SF-8 (mean±SD):  Role emotional (38.7±10.0)  Mental health (40.0±7.7)  Social functioning (42.6±9.4)  Vitality (45.0±8.3)  General health perception (43.2±9.9)  Role physical (47.8±7.0)  Physical functioning (47.7±7.1)  Bodily Pain (50.3±7.3)  Mental component summary (36.5±11.5)  Physical component summary (49.8±7.6) |
| Nahar et al., 2020 | Veterinary students, *n*=140, 92.9% female, mean age=25.88 (SD=3.75) years  Race/ethnicity:  White/Caucasian (87.9%)  Black/African American (0.7%)  American Indian or Alaska Native (0.7%)  Asian American (2.9%)  Hispanic American (4.3%)  Other (3.6%)  Academic Classification:  1^st^ year (25.0%)  2^nd^ year (30.7%)  3^rd^ year (26.4%)  4^th^ year (17.9%)  GPA:  2.00–2.49 (7.9%)  2.50–2.99 (19.3%)  3.0–3.49 (47.9%)  3.50–4.00 (24.3%)  Marital status:  Married (17.9%)  Single (80.0%)  Other (2.1%)  Children:  Yes (4.3%)  No (95.7%)  Work status:  Yes (11.4%)  No (88.6%)  Living arrangements:  On-Campus (5.0%)  Off-Campus (95.0%) | Demographic characteristics such as gender, age, academic classification, race/ethnicity, relationship status, and work status were collected  Additional characteristics were assessed such as perceived stress levels, anxiety, depression, current relaxation behavior practices, and Multi-Theory Model of Health constructs for initiation and sustenance of relaxation behavior | Demographic characteristics, stress management behaviors, and various parameters of mental health were inventoried to help determine the efficacy of the MTM in predicting initiation and sustenance of stress management behaviors among veterinary students  Constructs for initiation of health behavior change include participatory dialogue, behavioral confidence, and changes in the physical environment; constructs for sustenance of health behavior change include emotional transformation, practice for change, and changes in the social environment  Students’ current perceived stress levels were assessed using the perceived stress scale  Advantages and disadvantages of participatory dialogue were gauged with 5 items each, all were scored on a 5-point scale | Average stress level=21.64 (SD= 6.49; possible range 0–40)  Positive anxiety screen (60.0%)  Positive depression screen (32.9%)  For initiation of intentional relaxation behavior, 15.9% of the variance in initiation was explained by the lower-order terms forming the base model (*R*^2^=0.159, *P*<0.001); herein, significance was observed for depression (𝛽=−0.413, *P*=0.040) and academic classification (𝛽=−0.597, *P*<0.001)  Variance accounted for increased to 49.5% with the inclusion of MTM constructs in model 2 (*R*^2^=0.495, *P*<0.001) and in the final model, only behavioral confidence exhibited a significant association with initiation of relaxation behavior (𝛽=0.138, *P<*0.001)  From the initiation model, significant lower-order predictors included depression (*P*=0.040) and academic classification (*P*<0.001), while behavioral confidence was the only significant higher-order construct (*P*<0.001)  Regarding the sustenance model, significant lower-order predictors included perceived stress (*P*=0.009) and academic classification (*P*=0.001), while emotional transformation was the only significant higher-order construct (*P*<0.001)  Once all variables were accounted for in the model, emotional transformation held the only significant relationship to sustenance (𝛽=0.178, *P<*0.001) |
| Taylor et al., 2020 | First-year veterinary students, *n*=95 | Demographic characteristics including age and gender were collected  Prior to starting coursework, data was also collected regarding participants’ preexisting level of anxiety and/or depression | In addition to demographic characteristics, participants were assessed for depression and anxiety prior to starting coursework and immediately after completion of their first year of veterinary school  A modified version of the Hospital Anxiety and Depression Scale (HADS) was used to assess depression and anxiety; scores were categorized as either normal, borderline, or abnormal | Results indicated a mean difference of 2.29 (95% CI: 1.46-3.13) with a significant increase in anxiety, (*P*<0.01) when pre- and post-anxiety were analyzed  Depression, Pre- vs. Post-(%)  Abnormal (0.0% vs. 5.3%)  Borderline (4.2% vs. 10.5%)  Normal (95.8% vs. 84.2%)  Anxiety, Pre- vs. Post-(%)  Abnormal (33.7% vs. 60.0%)  Borderline (32.6% vs. 20.0%)  Normal (33.7% vs. 20.0%) |
| Wells et al., 2021 | Veterinary students, *n*=182, 85.7% female, mean age=25.2 (SD=2.98) years  Race/Ethnicity:  White/Caucasian (89%)  Non-White (7.1%)  Academic Classification: 1st year (34.1%)  2nd year (25.3%)  3rd year (26.4%)  4th year (11.0%)  Housing status: On Campus (3.3%) Off campus (93.4%)  Employment status:  Employed (19.2%)  Unemployed (77.5%)  GPA:  Less than 1.99 (0.5%)  2.00–2.49 (4.9%)  2.50–2.99 (15.9%)  3.00–3.49 (39.0%)  3.50–4.0 (34.6%) | Demographic characteristics such as gender, age, academic classification, race/ethnicity, housing, and employment status were collected  Additional data were collected including measures of perceived stress, as well as an assessment of anxiety, depression, and emotional intelligence (EI) | Data were obtained regarding general demographic characteristics as well as measures of stress, anxiety, depression (SAD), and emotional intelligence (EI)  EI, assessed using a 33-item scale, is a specific form of social intelligence that involves the capability to monitor and regulate personal feelings along with the feelings of others  Perceived stress was assessed using a 10-item scale (PSS)  Anxiety and depression were assessed using a 4-item questionnaire (PHQ-4) | More respondents screened positive for anxiety than negative (*n*=113 Anxiety; *n*=45 No anxiety), while more screened negative for depression than positive (*n*=97 No depression; *n*=61 Depression)  For anxiety, a mean difference of 5.29 was found between the EI scores of participants screening positive (M=115.31, SD=13.03) and negative (M=120.60, SD=13.09) (*P*=0.023)  For depression, a statistically significant, mean difference of 10.81 was calculated between participants screening positive (M=110.18, SD=11.21) or negative (M=120.99, SD=12.72) for depression (*P*<0.001)  Negative correlations were found between EI and stress (*r*=−0.417), EI and anxiety (*r*=−0.299), and EI and depression (*r*=−0.450) (*P*<0.001)  There were positive correlations between stress and anxiety (*r*=0.734), stress and depression (*r*=0.636), and anxiety and depression (*r*=0.590) (*P*<0.001)  After controlling for sociodemographic variables, EI was a significant predictor of stress (*b*=−0.239), anxiety (*b*=−0.044), and depression (*b*=−0.063) (*P*s*<*0.001) |
